# Prediction and Health Risk Assessment of Copper, Lead, Cadmium, Chromium, and Nickel in Tieguanyin Tea: A Case Study from Fujian, China

**DOI:** 10.3390/foods11111593

**Published:** 2022-05-28

**Authors:** Qinghua Yao, Minmin Huang, Yunyun Zheng, Meizhen Chen, Chongyao Huang, Qiu Lin

**Affiliations:** 1 Fujian Key Laboratory of Agro-Products Quality and Safety, Fuzhou 350003, China; hmmlwb@163.com (M.H.); yyzheng@xmu.edu.cn (Y.Z.); linqiu3163@163.com (Q.L.); 2 Institute of Quality Standards Testing Technology for Agro-Products, Fujian Academy of Agricultural Sciences, Fuzhou 350003, China; mijing.meizhen@163.com (M.C.); 18649787397@139.com (C.H.)

**Keywords:** tea, heavy metals, transfer rate, risk assessment, subpopulations

## Abstract

Heavy metal is widespread in food and the environment due to multiple origins, raising concerns over their persistent potential health risks. Contamination of multiple heavy metals in tea leaves is frequently reported. However, the dietary exposure risk that heavy metals in Tieguanyin tea (a famous type of oolong tea,) pose to different subpopulations has not been explored. In this study, contaminations of five heavy metals (Cu, Pb, Cr, Cd, and Ni) of concern in Tieguanyin tea were monitored, then the related health risk was assessed for six different subpopulations based on tea brewing experiments and a previous consumption survey. Results show that the mean levels found were as follows: 8.18 mg/kg (Cu), 0.84 mg/kg (Pb), 0.51 mg/kg (Cr), 0.04 mg/kg (Cd), and 1.90 mg/kg (Ni), respectively, and their transfer rates during tea brewing varied within 10.2–70.4%. All estimated daily intakes of individual targeted elements via Tieguanying tea consumption were far below their corresponding tolerable limits. The adjusted hazard index value ranged from 1.1 × 10^−2^ to 1.7 × 10^−2^, indicating that exposure to these five elements via drinking Tieguanyin tea would not pose significant non-cancer risks for six subpopulations under the current consumption habit. In addition, the carcinogenic risks associated with heavy metals (Pb, Cd, and Cr) were acceptable because no total cancer risk values exceeded the 10^−4^ threshold. However, in order to improve consumer protection, we still suggest that considerable attention should be paid to Pb, Ni, and Cd because of their high concentration in infusion, high extraction rate, and major carcinogenic risk contribution, respectively.

## 1. Introduction

Tea is a popular nonalcoholic beverage worldwide due to its refreshing taste and multiple medicinal properties. Based on the fermentation procedures and combination of sensory flavor, tea is classified as green, oolong, white, black, dark, and yellow tea [1]. Among these, oolong tea is subjected to the typical semi-fermentation processing procedure, including withering, shaking, firing, rolling, and drying [2,3]. As a typical oolong tea, Tieguanyin is one of China’s top ten famous teas and originated from Anxi county, Fujian, China around 300 years ago. Owing to the particular preparation, Tieguanyin tea is famous for its complex volatiles and substantial bioactivities [4,5] and has increasingly attracted consumer sand producers in recent years.

While the health benefits of tea have been investigated, tea also carries some potential risk factors which may pose a threat to the health of tea drinkers. Because of concern for environmental stability, difficult degradation, and easy accumulation, the risk associated with heavy metals has received increasing attention [6]. Chronic exposure to heavy metals may cause serious and irreversible damage such as cancer, memory deterioration, and bone fractures [7,8]. Tea plants can uptake heavy metals from fertilizers and soil, then accumulate in the leaves. Studies have also found that rainfall, dust, and the industrial equipment used for tea manufacturing could be other sources of metal elements [9,10]. Hence, tea can be easily contaminated by heavy metals, although their species and contamination levels are generally region-specific. It was reported that cadmium (Cd) concentration of green tea samples from China was higher compared to black tea and oolong tea, while black tea had higher copper (Cu) [11]. For tea samples from Turkey, the levels of Cu in green tea were higher compared to black tea [12]. Although some studies [13,14,15] were conducted to monitor the heavy metal contamination levels in tea and to assess the corresponding exposure risk for consumers, their outcome is not accurate enough as to the actual exposure to heavy metals. In general, tea infusion is the main way that we consume tea, so the released percentage of heavy metals should be considered for dietary risk assessment. Meanwhile, the released percentages of heavy metals from made tea to tea infusion vary with tea type. It was stated that the release of lead (Pb)and chromium (Cr) was much higher in black tea than in oolong or green tea [16]. Li et al. reported that infusion time is an important factor affecting element concentration in tea infusion [17]. Another important thing to note is that, while drinking tea is one potential source of heavy metal for consumers, this is not the same for every individual. The body weight and daily tea consumption per day of consumers are affected by demographic characteristics, such as gender, age, profession, and living area.

The tea industry is one of the dominant agricultural industries in Fujian, China. Residents there have a common tradition of drinking tea and are thought to consume the most Tieguanyin tea. Unfortunately, few reports are available on the accurate health risk assessment of heavy metal exposures to local people due to tea drinking. According to the pre-survey, this study therefore sought to monitor the distribution of Cu, Pb, Cr, Cd, and nickel (Ni) in Tieguanyin tea and to investigate their transfer behavior in brewing tea. Additionally, based on our previous tea consumption survey, the potential carcinogenic and non-carcinogenic risks for six different subpopulations (male/female, aged 18 to 40/aged over 41, and urban/rural) due to tea drinks were characterized. The results may provide data for risk communication and policymaking about tea quality control.

## 2. Materials and Methods

### 2.1. Regents and Materials

Nitric acid (HPLC grade) was obtained from Sinopharm Chemical Reagent Co., Ltd. (Shanghai, China) and standard solution of elements were purchased from Guobiao (Beijing) Testing & Certification Co., Ltd. (Beijing, China). Ninety-one Tieguanyin tea samples (500 g each) were randomly collected from tea shops, supermarkets, and tea factories in Fujian, China. Each tea sample was crushed, homogenized, and sifted through a 60-mesh (0.25 mmpore size) polyethylene sieve. Finally, all the sifted tea samples were stored in a dark and dry place until analysis.

All glassware and polyethylene bottles were carefully cleaned, then kept in the washing acid (10% HNO_3_), then rinsed with deionized water and air-dried on a clean test bench before use.

### 2.2. Tea Brewing and Sample Digestion

Tea infusion was prepared as follows: 7 g of made tea was poured into 150 mL boiling deionized water for 10 min. Subsequently, the water extract was filtered and cooled down to room temperature. Prior to inductively coupled plasma mass spectrometry (ICP-MS) analysis, the tea infusion was acidified to obtain a 0.2% HNO_3_ solution and filtered. On the other hand, each sifted tea sample (0.3 g) was weighed into a Teflon digestion vessel and treated overnight with 5 mL HNO_3_, then digested in a microwave digester (TOPEX, Preekem Instrument Technology Co., Ltd., Shanghai, China). The digestion parameters are summarized in Table 1. After digestion, the digested solution was evaporated to remove HNO_3_, then cooled to room temperature. The digested solution was further made up to volume with 0.2% HNO_3_ solution and filtered before measurement.

The concentrations of Cu, Pb, Cr, Cd, and Ni in tea samples and tea infusion were determined by ICP-MS (XSERIES 2, Thermo Fisher Scientific Co., Ltd., Waltham, MA, USA). The working conditions of ICP-MS are shown in Table 2. The validity of the digestion procedure and ICP-MS instrument performance were evaluated using the certified reference material (CRM) of green tea (GBW10052, provided by the Institute of Geophysical and Geochemical Sciences, Beijing, China). For tea infusion, three spiked mixtures of related heavy metals with concentrations of 10, 50, and 100 μg/L were prepared and analyzed for quality assurance and quality control. As presented in Table 3, the recoveries of studied elements indicated that the accuracy and precision of the proposed methods were acceptable. Linearity values of all calibration curves were satisfactory (R^2^ = 0.999). The limits of detection (LODs) were defined as three times the standard deviation (SD) of 10 reagent blanks including 1% HNO_3_. For Cu, Pb, Cr, Cd, and Ni, the LOD was 0.09, 0.01, 0.009, 0.003, and 0.02 μg/L, respectively. Ten times the SD was regarded as the limit of quantification (LOQs). The LOQs of these elements were as follows: 0.3 μg/L (Cu), 0.03 μg/L (Pb), 0.03 μg/L (Cr), 0.01 μg/L (Cd), and 0.06 μg/L (Ni), respectively. All samples were analyzed in triplicate. Undetected samples were assigned the value of corresponding LOQ.

### 2.3. Data Analysis and Health Risk Assessment

The transfer rate *T* (%) of the target element from made tea into tea infusion is calculated via the following Equation (1):

(1) $$T\;(\%)=\frac{C_{i}\times V_{i}}{C_{t}\times M_{t}}\times 100\%$$

where *C_i_* and *C_t_* are the concentrations of each metal element in the tea infusion and made tea, respectively. *V_i_* is the volume of tea infusion (150 mL), and *M_t_* refers to the quantity of tea sample used for brewing (7 g).

The non-carcinogenic hazard quotient (*HQ*) via drinking tea infusion was assessed via Equations (2) and (3), dividing the estimated daily intake (*EDI*) by the reference dose (*RfD*) [18].

(2) $$\mathrm{EDI}=\frac{C\times D\times T}{B_{W}}$$

(3) $$\mathrm{HQ}=\frac{EDI}{RfD}\;$$

where *C* refers to the concentration of the target heavy metal element, *D* represents daily tea consumption amount, *T* is the transfer rate, and *B_w_* is the body weight. The data of daily tea consumption amount and the bodyweight of different subpopulations were obtained from our previous survey [19]. If the *HQ* value was less than one, it was assumed that the non-carcinogenic risk would be acceptable. Higher *HQ* values indicate higher risk.

The hazard index (*HI*),the summation of each *HQ*, was used to express the overall potential risk of the exposure to multiple contaminants. It was calculated using Equation (4) established by USEPA [20].

(4) $$HI=\overset{n}{\underset{i=1}{\sum }}R_{i}^{n}$$

Targeted carcinogenic risk (*TR*) indicates the probability of an individual to develop cancer due to heavy metal exposure. It was calculated using Equation (5).

*TR* = *EDI* × *Sf* (5)

where *Sf* is the oral carcinogenic slope factor. The total cancer risk (*TR_total_*) was the sum of the TR value.

## 3. Results and Discussion

### 3.1. Copper, Lead, Cadmium, Chromium, and Nickel in Tieguanyin Tea

The contamination levels of the five targeted metals in Tieguanyin tea samples are shown in Table 4. The highest levels of Cu, Pb, Cr, Cd, and Ni detected in samples were 11.61 mg/kg, 2.00 mg/kg, 1.38 mg/kg, 0.11 mg/kg, and 2.93 mg/kg. The mean levels of these metals were 8.18 mg/kg for Cu, 0.84 mg/kg for Pb, 0.51 mg/kg for Cr, 0.04 mg/kg for Cd, and 1.90 mg/kg for Ni, respectively. Wide ranges of heavy metal levels were observed, indicating that numerous effects contributed to the contents of metals in tea. Previous studies reported that the contents of trace elements in tea depend on the age of tea leaves when collected, soil quality, rainfall incidence, or altitude of tea plantation, and manufacturing processes, such as equipment used for withering, rolling, fermentation, or drying [10,21].

Cu is one of the most abundant trace elements in tea leaves [16]. It is considered as an essential element for human health at low concentration [25], but excessive intake can cause anemia, developmental retardation, and metabolic disorders [26]. Our study showed that the Cu content in Tieguanyin tea is lower than that in Puerh tea [13]. However, it was much higher than that reported by Shen & Chen [16]. They reported that average concentration of Cu in green tea, oolong tea, and black tea was 0.4 mg/kg, 0.9 mg/kg, and 0.3 mg/kg, respectively. A previous study showed that rolling by rotorvane is the key stage affecting the Cu levels in the final tea samples because the extruded viscous succus may increase the possibility of Cu adherence to the tea leaf surface [10]. Hence, further study is needed to find out the source of Cu in Tieguanyin tea.

Excess exposure to soil and vegetables polluted with Pb and Cd can decrease human life expectance by 9–0 years [27]. Cao et al. found the average Pb and Cd concentration to be 0.57 mg/kg and 0.018 mg/kg for fermented Puerh tea, while 0.35 mg/kg and 0.032 mg/kg for raw Puerh tea [13]. Falahi and Hedaiati measured the average Pb and Cd contents in black tea commercialized in Iran at 8.2 mg/kg and 0.21 mg/kg [28]. Li et al. reported that Pb contents in 26 green tea samples from China ranged from 0.12 mg/kg to 2.24 mg/kg with a mean value of 0.92 mg/kg, and Cd contents varied from 0.025 mg/kg to 0.11 mg/kg with a mean value of 0.055 mg/kg [17]. Compared with these previous studies, our results regarded that the contents of Pb in Tieguanyin tea were similar to those found in Puerh tea and green tea but much lower than those found in black tea.

In the environment, Cr is present in several forms, such as chromium(0), (II), (III), and (VI) [29]. Among these, the health effects of Cr^3+^ and Cr^6+^ are commonly studied. Although Cr^6+^ is much more toxic than Cr^3+^, the total chromium was analyzed for biological samples because of the biological reduction of Cr^6+^ toCr^3+^ [29,30]. The mean Cr concentration detected in Tieguanyin tea samples was in accordance with those in oolong tea from China (0.4 mg/kg) [11,16] and black tea from Mexico (0.4 mg/kg) [31].A higher mean of the Cr level was reported in the green tea from Jiangxi of China with 1.6 mg/kg [17] and Thailand with 1.5 mg/kg [32], and in the Puerh tea from Yunnan of China with 3.9 mg/kg [14]. Much lower Cr content at an average level of 0.01 mg/kg was recorded, however, in green tea collected by Shen and Chen [16].

Human activities such as mining and agriculture cause extensive localized pollution with Ni throughout the world [33]. Exposure to Ni beyond a certain threshold concentration may lead to some health problems, e.g., skin allergies, lung fibrosis, and cardiovascular system poisoning [34]. The Tieguanyin tea Ni concentration in our study was in line with there port of Seenivasan et al. [35], while slightly lower than that of puerh tea from southwest China (6.27 mg/kg) [36], green tea from Jiangxi of China (7.71 mg/kg) [17], and Turkish tea (23.3 mg/kg) [37].

### 3.2. Transfer Rate of Targeted Elements during Tea Brewing

**Figure 1:**
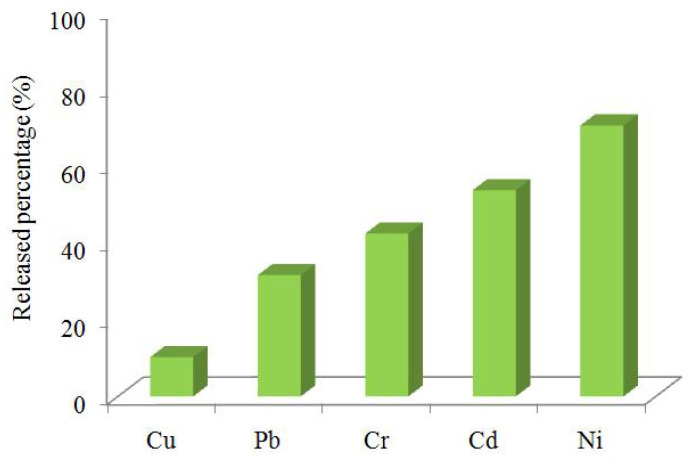
Released percentage of Cu, Pb, Cr, Cd, and Ni during tea brewing.

The elemental solubility during tea brewing was explored to assess the actual exposure amount for consumers via drinking this beverage. It could be expected that the elemental contents in the infusions were lower than those in made tea. As shown in Figure 1, the mean transfer rate of these five targeted elements can be arranged in the following order with an increasing tendency: Cu (10.2%), Pb (31.5%), Cr (42.3%), Cd (53.6%), and Ni (70.4%).The differences of element transfer rates could be principally explained by whether the compound in made tea is more soluble during brewing or strongly bound to the matrix [10]. The transfer rate of Cu in our study was lower than results obtained from Shen and Chen (22.9% for green tea, 23.9%foroolong tea, and21.8%forblack tea) [16]. The low leaching rate of Cu here could be associated with the strong connection between Cu and some matrix in Tieguanyin tea. The transfer rate for Pb was higher for Tieguanyin tea compared to the green tea (7.1%), while lower than that in oolong tea (50.1%) and black tea (58.6%) [16]. Although Pb in Tieguanyin tea was moderately extracted, Pb concentration in some tea infusion could still exceed the maximum allowable limit of 0.01 mg/L set for drinking water [38]. Therefore, the source and control practices for Pb in tea remain a concern. For Cr, the mean transfer rate observed in Tieguanyin tea was close to some previous reports. Shen and Chen found that the Cr transfer rate in oolong tea is 47.5% [16]. Natesan and Rangana than reported that the percentage transfer of Cr was 42.2% for black tea [39]. Although 53.6% of the total Cd was released to the tea infusion, Cd in the tea infusion was generally low—even below the detectable limit for several samples. Extremely low concentration of Cd in tea infusion could be also found in the existing literature. Shen and Chen reported that the mean Cd concentration was 0.05 μg/L for eighteen oolong tea infusion samples, 0.6µg/L for fifteen black tea infusion samples [16]. The analyzed results for 10 black tea infusion samples showed that the Cd level ranged from below the detectable limit to 1.093 mg/L [40]. Among the five targeted elements in the present study, Ni has the highest transfer rate. A high transfer rate of Ni was also observed in black tea at 74.9% [39]. Similar findings were obtained by Zhang et al. [10] and Li et al. [17], which illustrated that the total transfer rate of Ni was 75.7% for black tea and 82.4% for green tea. In general, Ni was considered to be a highly extractable element in tea leaves [41].

### 3.3. Daily Intake Estimation and Risk Assessment

Non-cancer risk of these elements via drinking tea infusions for consumers was assessed by calculating HQ. The HQ values are summarized in Table 5. HQ for individual metal exposure was much lower than 1%. The results suggested that drinking tea infusions in daily life exhibited an extremely low portion of *RfD* and would be unlikely to pose a significant health risk. It was consistent with many former reports [10,17]. Due to different drinking habits, males, adults over the age of 41, and urban residents have higher exposure risk than females, adults over the age of 18 but under 40, and rural residents, respectively. The potential health risk of Pb and Cuwere found to be the highest and lowest, respectively. In contrast, HQ values of metal exposure were in the order of Cu > Ni > Pb > Cd > Crvia drinking green tea [17] and Cu > Pb > Cd via drinking Puerh tea [13]. This indicated that more attention should be focused on the Pb in Tieguanyin tea.

HI values much less than 1 implied that the overall non-cancer risk of multiple metals via drinking Tieguanyin tea infusion was within the safety boundaries. This finding is consistent with the results from Shen and Chen who found that the HI values of green tea, oolong tea, and black tea consumption ranged from 1.3 × 10^−3^ to 6.7 × 10^−1^ [16]. However, relatively high HI values of tea consumption were described in some published works. Cao et al. reported that HI values of Puerh tea consumption for the consumers in Kunming City and Puerh City were 0.17 and 0.29, respectively [13]. Li et al. conducted a study in the Jiangxi Province of China and showed that HI value of drinking green tea infusion was 0.306 [17]. In a study from Ghana, the HI value even exceeded 1 without considering the transfer rate of elements during tea brewing [9]. Compared with rice, fruits, and vegetables, HI values here were lower than that from some previous studies [42,43].

In addition, the bio accessibility and bioavailability of the targeted contaminants were also considered in this study. Tey could be expressed as the absorption percentages of their corresponding *RfDs* when calculating adjusted HQs [44]. According to previous reports, the bioavailability of Cu, Pb, Cr, Cd, and Ni were 40%, 60%, 1%, 50%, and 10%, respectively [45,46,47,48,49]. As a result, the adjusted HQ for consumers was lower than the respective HQ. Moreover, the adjusted HI values for different subpopulations decreased by approximately 20% compared with the former values. This reveals that the combined effect of these five elements in Tieguanyintea infusions would not pose significant non-cancer risks to consumer health under the populations’ current consumption habits.

**Figure 2:**
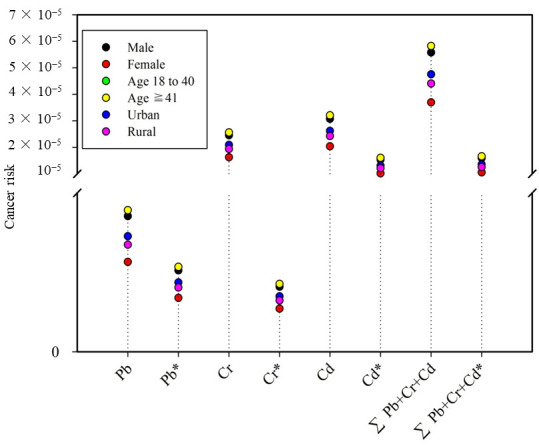
Cancer risk for different subpopulations due to drink tea infusion. Note: * means that the bioavailability of element was considered.

As previously stated, three elements (Pb, Cd, and Cr) in this study have potential carcinogenicity, and their oral cancer slopes have been established (Table 4). The TR values range from 1.62 × 10^−7^ (in Cr for females) to 1.60 × 10^−5^ (in Cd for adults over the age of 41) (Figure 2), which is lower than the upper limit for the acceptable carcinogenic risk (one in ten-thousand) given by USEPA [50]. By summing the individual TR values, the TR_total_ value was calculated. No TR_total_ values for six different subpopulations exceeded the 10^−4^ threshold. Compared with other foodstuffs, it was apparent that carcinogenic risks associated with heavy metals (Pb, Cd, and Cr) through drinking Tiguanyin tea infusions was acceptable. Castro-González et al. found that the combined effect of heavy metals in cow’s milk would pose a serious carcinogenic risk for some consumers in Mexico [51]. In another study, Chai et al. showed the carcinogenic risk posed by bioaccessible heavy metals in shellfish from several Chinese cities was 10-fold higher than the upper limit (10^−4^) [52]. Therefore, based on the results in the present study, drinking the Tieguanyin tea infusions have an acceptable potential for carcinogenic risk attributable to Pb, Cd, and Cr. However, Cd contamination in Tieguanyin tea should remain of concern, as it was the major carcinogenic risk contributor with an approximate percentage of 97%.

### 3.4. Uncertainty and Prospect

Although this study was carefully designed and the researchers strived for quality results, there are inevitably some limitations from insufficient knowledge. Admittedly, uncertainties in the contamination data could be reduced by collecting more samples. Consequently, it was an extremely expensive approach [53]. In addition, various forms of these metals and their respective toxicities have not been considered here. Determination of total metal concentrations may seem to be over- or under-estimates of the real exposure to these toxic metals. On the other hand, the uncertainties in the consumption data might have led also to an over- or under-estimation, as discussed in our previous study [19]. Besides, we should emphasize that tea consumption was not the only exposure pathway of heavy metals in human daily diets; many studies have reported that other foods, such as vegetables [43], shellfish [52], meat [53], rice [42], milk [51], and even water, were also significant exposure sources [54]. These uncertainties should be considered when making further management or performing future research.

## 4. Conclusions

The current study investigated the occurrence of Cu, Pb, Cr, Cd, and Ni in Tieguanyin tea collected from Fujian province, and firstly examined their transfer rates during tea brewing. Wide concentration ranges were observed for these metals with the average level in order of Cu, Ni, Pb, Cr, and Cd, respectively. The released percentage of those targeted elements decreased in the order of Ni, Cd, Cr, Pb, and Cu. Although both non-cancer risk and carcinogenic risk from each individual element or multiple elements via Tieguanyin tea consumptions for six different are acceptable, considerable attention should also be paid to Pb, Ni, and Cd due to high concentration in infusion, high transfer rate during tea brewing, and major carcinogenic risk contribution, respectively. This work highlights the importance of establishing and implementing good agricultural practices (GAP), risk management measures, and food safety education programs for stakeholders such as the tea industry, food safety agencies, and consumers.

## Figures and Tables

**Table 1:** Working conditions of microwave digestion instrument.

Steps	Temperature (°C)	Time (min)
1	120	5
2	160	10
3	180	10

**Table 2:** Optimized operating for ICP-MS.

Parameters	Value	Parameters	Value
Radio frequency power	1.2 kw	Isotopes monitored	^52^Cr
Plasma gas	8.0 L/min		^60^Ni
Auxiliary gas	1.1 L/min		^65^Cu
Carrier gas	0.7 L/min		^114^Cd
Collision gas	6.0 L/min		^208^Pb

**Table 3:** LOD, LOQ, recovery, and the performance of the analytical method.

Element	Certified Reference Tea (Mean ± SD) (μg kg^−1^)		Recovery for Tea Infusion (%)			LOD (μg L^−1^)	LOQ (μg L^−1^)	R^2^
	Certified Value	Detected Value	10 μg L^−^^1^	50 μg L^−^^1^	100 μg L^−^^1^			
Cu	24 ± 1	24.4 ± 0.5	110.6	110.7	114.2	0.09	0.3	0.999
Ni	5.4 ± 0.4	5.4 ± 0.2	103.6	96.8	98.8	0.02	0.06	0.999
Pb	1.6 ± 0.2	1.7 ± 0.1	90.1	85.9	87.2	0.01	0.03	0.999
Cd	0.076 ± 0.004	0.077 ± 0.001	85.3	87.3	84.3	0.003	0.01	0.999
Cr	0.92 ± 0.20	0.92 ± 0.01	100.4	94.7	96.8	0.009	0.03	0.999

**Table 4:** The contamination levels, oral reference dose (*RfD*) values and slope factors (*Sf*) of five potentiality toxic elements (PTEs).

	Range (mg kg^−1^)	Mean ± SD	*RfD* (μg kg^−1^ bw day^−1^) ^a^	*Sf* (mg kg^−1^ bw Day^−1^) ^b^
Cu	2.65–11.61	8.18 ± 2.10	40	
Pb	0.21–2.00	0.84 ± 0.40	3.57	0.0085
Cr	0.08–1.38	0.51 ± 0.28	3	0.5
Cd	ND-0.11	0.04 ± 0.02	1	6.3
Ni	1.02–2.93	1.90 ± 0.70	20	

^a^ The *RfD* of Pb adapted from Yao et al. [15]; others adapted from USEPA [22]. ^b^ The *Sf* values of Cd adapted from Bamuwamye et al. [23]; others adapted from the Integrated Risk Information System of USEPA [24].

**Table 5:** Non-carcinogenic hazard quotient of heavy metals due to tea infusion consumption.

	HQ (%)						Adjusted HQ (%) ^a^					
	Male	Female	Urban	Rural	Age 18–40	Age ≥ 41	Male	Female	Urban	Rural	Age 18–40	Age ≥ 41
Cu	4.7 × 10^−3^	3.1 × 10^−3^	4.0 × 10^−3^	3.7 × 10^−3^	3.7 × 10^−3^	5.0 × 10 ^3^	2.0 × 10^−3^	1.2 × 10^−3^	1.6 × 10^−3^	1.5 × 10^−3^	1.5 × 10^−3^	2.0 × 10^−3^
Pb	1.7 × 10^−2^	1.1 × 10^−2^	1.4 × 10^−2^	1.3 × 10^−2^	1.3 × 10^−2^	1.8 × 10^−2^	1.0 × 10^−2^	6.7 × 10^−3^	8.6 × 10^−3^	8.0 × 10^−3^	8.0 × 10^−3^	1.0 × 10^−2^
Cr	1.6 × 10^−2^	1.1 × 10^−2^	1.4 × 10^−2^	1.3 × 10^−2^	1.3 × 10^−2^	1.7 × 10^−2^	1.6 × 10^−4^	1.1 × 10^−4^	1.4 × 10^−4^	1.3 × 10^−4^	1.3 × 10^−4^	1.7 × 10^−4^
Cd	4.9 × 10^−3^	3.2 × 10^−3^	4.2 × 10^−3^	3.8 × 10^−3^	3.8 × 10^−3^	5.1 × 10^−3^	2.4 × 10^−3^	1.6 × 10^−3^	2.1 × 10^−3^	1.9 × 10^−3^	1.9 × 10^−3^	2.5 × 10^−3^
Ni	1.5 × 10^−2^	1.0 × 10^−2^	1.3 × 10^−2^	1.2 × 10^−2^	1.2 × 10^−2^	1.6 × 10^−2^	1.5 × 10^−3^	1.0 × 10^−3^	1.3 × 10^−3^	1.2 × 10^−3^	1.2 × 10^−3^	1.6 × 10^−3^
***HI***	5.8 × 10^−2^	3.8 × 10^−2^	4.9 × 10^−2^	4.6 × 10^−2^	4.6 × 10^−2^	6.1 × 10^−2^	1.6 × 10^−2^	1.1 × 10^−2^	1.4 × 10^−2^	1.3 × 10^−2^	1.3 × 10^−2^	1.7 × 10^−2^

^a^ Adjusted HQ was calculated as HQ multiplying by the bioavailability of the corresponding element.

## Data Availability

The data presented in this study are available in article.
